# A Unique Interaction of Methotrexate and Nitrofurantoin Resulting in Irreversible Pulmonary Fibrosis

**DOI:** 10.7759/cureus.20892

**Published:** 2022-01-03

**Authors:** Smriti Kochhar, Veera Jayasree Latha Bommu, Mariusz Kocur, Viraj Shah, Pramil Cheriyath, Thomas Lake

**Affiliations:** 1 Medicine, Hackensack Meridian Health, Ocean University Medical Center, Brick, USA; 2 Medicine, Rowan University School of Osteopathic Medicine, Stratford, USA; 3 Internal Medicine, Hackensack Meridian Health, Ocean University Medical Center, Brick, USA; 4 Colorectal Surgery, Hackensack Meridian Health, Ocean University Medical Center, Brick, USA

**Keywords:** drug-drug interaction, rheumatoid arthritis, restrictive lung disease, pulmonary fibrosis, nitrofurantoin, methotrexate

## Abstract

Pulmonary toxicity is the most well-known severe complication related to both methotrexate and nitrofurantoin, which can present as acute, subacute, and chronic. Rheumatoid arthritis is also known to cause pulmonary disease if left untreated. In this report, we present a unique case of a 94-year-old female being treated with methotrexate for several years and then treated with nitrofurantoin in the setting of rheumatoid arthritis and chronic urinary tract infections, resulting in irreversible pulmonary fibrosis, which can further cause more susceptibility to infections and pneumonia. Drug-drug interactions are common in polypharmacy and a patient's history should be analyzed thoroughly before prescribing any new medication that can cause more harm to the patient than good.

## Introduction

In 30-40% of individuals with rheumatoid arthritis (RA), radiographic or pulmonary function abnormalities suggestive of interstitial fibrosis or restrictive lung disease are present [1]. Pulmonary toxicity is the most well-known severe reaction of nitrofurantoin, which can present as acute, subacute, and chronic. Fever, chills, cough, myalgia, and dyspnea are all symptoms of acute pulmonary response syndrome, which can resemble the presentation of acute interstitial pneumonia (AIP) [2]. Pulmonary fibrosis is a rare complication of methotrexate (MTX) whereas hepatotoxicity, nausea, fatigue, alopecia, oral ulcers, and bone marrow suppression are relatively more common [3]. AIP and acute exacerbations of idiopathic pulmonary fibrosis (AEIPF) are respiratory illnesses marked by the rapid progression of dyspnea and cough [4]. This is a second case report of the interaction of methotrexate and nitrofurantoin resulting in irreversible pulmonary fibrosis [5] in a 94-year-old female with RA.

## Case presentation

A 94-year-old female presented with a chief complaint of shortness of breath that started a couple of weeks ago in September 2021 with a non-productive cough. Six weeks earlier, she was treated for pneumonia at the nursing center with antibiotics. The patient was being treated for RA with MTX for more than 10 years. She was also on nitrofurantoin daily for urinary tract infection suppression since 2017. Her past medical history includes elevated cholesterol and hypertension. Her home medications includes atorvastatin (10 mg), hydroxychloroquine (200 mg), nitrofurantoin (50mg), omeprazole (20 mg), oxybutynin (10mg) , sertraline (100 mg). She had been completely vaccinated against COVID-19. She was a lifetime non-smoker. Her vital signs at intake were 96.5 beats per minute pulse, 20 breaths per minute respiration, 141/79 mmHg blood pressure, 98% pulse oximetry with 2L nasal cannula. Her physical exam was unremarkable other than the presence of rhonchi in bilateral lungs.

On complete blood count she had an increase in neutrophils and monocytes at 76.9% and 10.9% respectively, with a decrease in lymphocytes at 9%; her WBC count was 9.1 x10^3^/UL (4.5-11 x 10^3^/UL). Her comprehensive metabolic panel showed low sodium at 130 mmol/dL, albumin at 3.3 g/dL, and increased troponin of 0.08 ng/mL. On viral serology she tested positive for rhinovirus/enterovirus. Her ECG showed sinus with first degree atrioventricular block and left anterior fascicular block.

**Figure 1:**
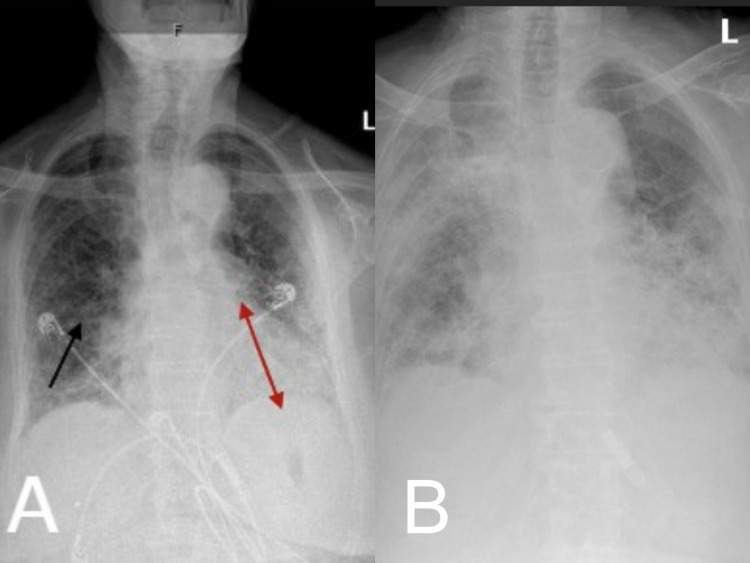
Chest x-ray: (A) showing lobar pneumonia (area under the double headed arrow); (B): showing diffuse pneumonia on hospital day 13.

**Figure 2:**
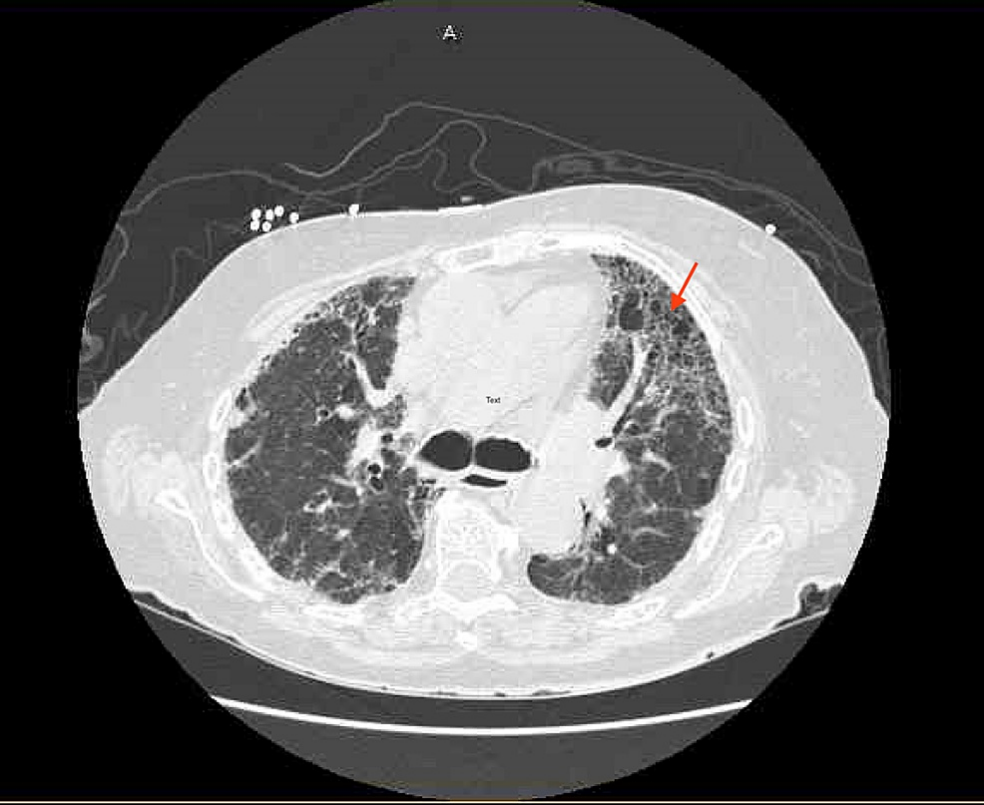
Chest CT without contrast showing honeycombing-lesion of pulmonary fibrosis (red arrow).

**Figure 3:**
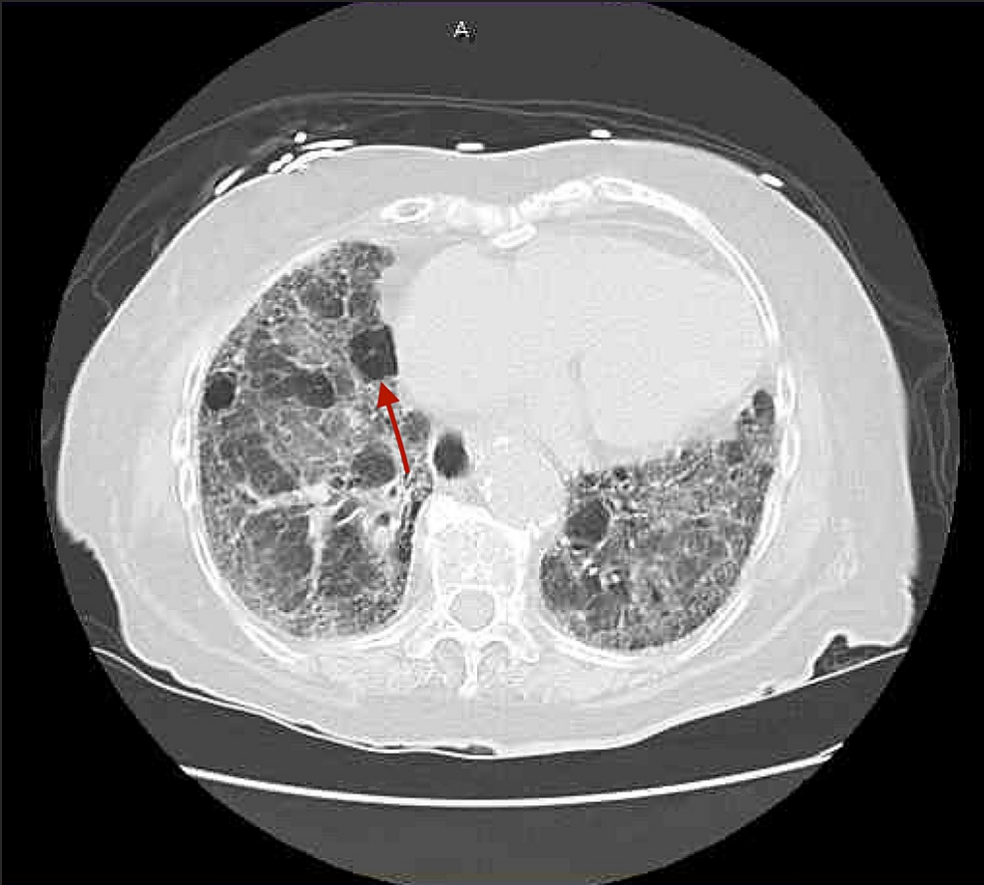
Chest CT without contrast showing air spaces in the lungs (red arrow).

Her portable chest x-ray showed bilateral mid and lower lung zone airspace disease, increased bilateral vascular congestive markings with infectious or viral or inflammatory processes are suspected (Figures 1A-1B). Computed tomography (CT) of the chest without contrast revealed moderate honeycombing pattern- lesion of pulmonary fibrosis and air spaces in the lungs (Figures 2-3). She was treated for pneumonia with ceftriaxone and azithromycin but even after the resolution of pneumonia, she had difficulty breathing and elevated neutrophils at 77.4% and lymphocytes at 8.1%. Her hematocrit, RBC, and hemoglobin dropped to 29.4%, 3.19 million/mm^3^, and 9.6g/dl while her WBC increased to 11.2 x10^3^/UL. Her most recent pulse oximetry also showed 97% on 2L nasal cannula. On hospital day 13, she again tested positive for rhinovirus/enterovirus with an increase of WBC from 9.6 x10^3^ /UL to 14 x10^3^/UL. After this, she refused treatment and was on hospice and passed away after a few days.

## Discussion

In 1953, the FDA approved nitrofurantoin for the treatment of lower urinary tract infection, and it was widely prescribed for the next two decades until it was superseded by trimethoprim/sulfamethoxazole and beta-lactam antibiotics in the 1970s [6].

The presence of bacterial nitroreductases, which convert nitrofurantoin to highly reactive electrophilic intermediates, corresponds with nitrofurantoin susceptibility in bacteria. These intermediates have been found to target bacterial ribosomal proteins in a non-specific manner, halting protein synthesis completely [7]. Hainer et al. revealed the production of toxic metabolic products of nitrofurantoin in the presence of oxygen and lung microsomes may cause lung injury and thus result in diffuse interstitial lung fibrosis. This explains the prevalence of a chronic pulmonary reaction in the elderly [8]. Even among patients for whom nitrofurantoin has traditionally been discouraged, such as men and the elderly, large population-level datasets show a high reliance on nitrofurantoin [9].

Many medications are only successful in 25-60% of patients, and there are more than two million incidents of adverse drug reactions in the United States each year, with 100,000 deaths [10]. Pulmonary toxicity is the most well-known severe reaction of nitrofurantoin. Nitrofurantoin-induced lung toxicity can be divided into three categories: acute, subacute, and chronic pulmonary responses. Fever, chills, cough, myalgia, and dyspnea are all symptoms of acute pulmonary response syndrome. Sub-acute pulmonary responses, which are characterized by a persistent dry cough, dyspnea, and fever, can also develop. The insidious onset of persistent dry cough and dyspnea is linked to this chronic pulmonary response. Acute, subacute, and chronic pulmonary toxicity can all be reversed by stopping the medicine immediately. This effect is still rare, according to one study, which found that the calculated frequency for all pulmonary reactions was only present in 0.001% of nitrofurantoin courses. Hepatic responses such as cholestatic jaundice, hepatitis, and hepatic necrosis are also unusual side effects. In these circumstances, the medicine should be stopped right away. Another unusual side effect is peripheral neuropathy, which is most commonly associated with long-term use in patients with poor renal function [2]. 

Patients with RA, juvenile RA, hematological malignancies, and skin disorders are treated with MTX (e.g., psoriasis, systemic sclerosis, dermatomyositis, cutaneous sarcoidosis, morphea, and different types of eczema). The antineoplastic/anti-rheumatic effects of MTX, as well as its toxicity, can be explained by the depletion of folate and the cells' inability to synthesize DNA at the same time [11]. Because 50-70% of MTX is bound to serum albumin for distribution, it can be displaced by other medications given at the same time. Non-steroidal anti-inflammatory drugs (NSAIDs) and disease-modifying antirheumatic drugs (DMARDs) may impair MTX clearance in the kidneys, whereas the risk of liver damage and bone marrow suppression is enhanced when using medications with similar adverse drug reactions (ADRs) [12].

AIP and AEIPF are respiratory illnesses marked by the rapid progression of dyspnea and cough. Those with AEIPF have prior idiopathic pulmonary fibrosis, but patients with AIP do not have any underlying diseases [4]. Our patient has a history of RA that has been under remission with MTX for many years and she has been on 50mg of nitrofurantoin daily for the last four years for the suppression of urinary tract infection. The patient developed fever, chills, non-productive cough, and shortness of breath certainly due to the interaction between MTX and nitrofurantoin that has aggravated the underlying pulmonary fibrosis. This resulted in the development of pneumonia that was evidenced by bilateral mid and lower lung zone airspace disease and increased bilateral vascular congestive markings found on the portable chest x-ray.

While cytotoxic chemotherapeutic agents, such as MTX, are the most common cause of interstitial lung disease, early high-resolution CT scans may show only scattered or diffuse areas of ground-glass opacity. Progression of the disease can be noted by findings of fibrosis (traction bronchiectasis, honeycombing) on the CT scan predominantly in the basal distribution that leads to subpleural cystic airspaces with thick walls that are known as “honeycombing” [10]. She was treated for pneumonia with ceftriaxone and azithromycin but even after the resolution of pneumonia, she had difficulty breathing and elevated neutrophils at 77.4% and lymphocytes at 8.1% and her WBC count stayed elevated even after completing the course of antibiotics.

The first case of interaction of MTX and nitrofurantoin was reported by Gowarty JL et al., in a patient with non-Hodgkin's lymphoma of the central nervous system receiving high-dose MTX while also receiving nitrofurantoin for a urinary tract infection. The patient’s initial MTX clearance was subsequently affected by the addition of nitrofurantoin, but it recovered to baseline when the nitrofurantoin was stopped [5]. Our patient refused further treatment and was on hospice care and later succumbed to death.

In human dermal fibroblasts, hydroxychloroquine efficiently decreased fibroblast proliferation, suppressed metabolic activities in fibrotic skin disorders, and inhibited extracellular signaling-regulated kinase ERK1/2 activation [13]. Hence, hydroxychloroquine can be used in place of MTX as it is protective of pulmonary fibrosis. Idiopathic pulmonary fibrosis (IPF) is a debilitating disease with no known cause, marked by permanent morphological changes that eventually lead to lung fibrosis and death [14]. Pneumonia in patients with IPF can further exacerbate the condition resulting in death. So, it is crucial to treat pneumonia in these patients aggressively.

Though the disease and the treatment both are related to the same side effects, we need to find a better way to monitor the side effects of these medications. Cottin et al., in a 124-patient clinical trial, was able to prove that there was no benefit of periodic pulmonary function tests as they did not allow us to detect MTX-induced pneumonitis before clinical symptoms [15]. Physicians should check the patient's history and family history to rule out any lung conditions to prevent doing any harm to the patient. At the same time, when prescribing these medications, patients should be educated to contact their primary care physician when they notice any change in breathing patterns until a better test or drug comes.

## Conclusions

MTX is known to cause many drug-to-drug interactions. However, the interaction of MTX and nitrofurantoin is unique and is rarely reported. Diagnostic work-up should include examination, laboratory studies, chest radiography, and high-resolution CT of the chest and pulmonary function testing. Physicians should be aware of the deadly nature of this interaction before prescribing and can go for other alternative medications such as hydroxychloroquine, which is supposed to protect against pulmonary fibrosis. Pneumonia in patients with underlying irreversible pulmonary fibrosis should be treated aggressively to prevent mortality.
